# Audit of Surgical Complications Post-limb Salvage Procedures for Aggressive Bone Tumours at a Developing Tumour Unit

**DOI:** 10.7759/cureus.117459

**Published:** 2026-10-05

**Authors:** Marule Paul Kgagudi, Maxwell Jingo, Thulani Ntshangase, Tiego Hlapolosa

**Affiliations:** 1 Orthopaedic Surgery, Clinical Medicine, Faculty of Health Sciences, Wits University, Johannesburg, ZAF; 2 Orthopaedic Surgery, University of Limpopo, Polokwane, ZAF

**Keywords:** aggressive bone tumours, complications, limb-salvage procedure, primary malignant bone tumours, prosthetic joint infection

## Abstract

Background

The incidence of malignant bone tumours remains relatively low in our setting. However, our patients typically present late with large tumour volumes, making limb-salvage procedures difficult and prone to post-operative complications. Due to a paucity of literature in a clinical context similar to ours, we elected to assess complications experienced by our cohort of patients.

Aim & setting

We aimed to study complications experienced by our patient cohort post-limb salvage and tumour prosthesis replacement for aggressive bone tumours. This study was set at the Chris Hani Baragwanath Academic Hospital Teaching Hospital in Johannesburg from January 2017 to December 2024.

Methods

We conducted a retrospective review of records of patients who underwent limb salvage for aggressive musculoskeletal tumours at our hospital. Patients with aggressive bone tumours treated at a different unit and those who underwent revision surgery were excluded from the study. Demographic data, pathology factors, and surgery factors were extracted for analysis. Descriptive statistics were computed using Stata 18 (StataCorp LLC, College Station, Texas).

Results

A total of 20 patient records met the inclusion criteria for review. The majority of the patients were of African descent, with all age groups having an equal distribution of the burden of disease: five (0-17 years), five (18-24 years), five (25-34 years), and five (> 35 years). Osteosarcoma accounted for 45% (9/20) of diagnoses, followed by giant cell tumour (GCT) of bone. Our cohort showed a significant predilection for lower-limb involvement, with the latter accounting for 90%(18/20) of cases. Seventy percent of our patients experienced complications, with a majority of complications being prosthesis-related, such as prosthetic joint infection (PJIs) and instability.

Conclusion

The use of limb-salvage surgery with tumour prosthesis implants for malignant bone tumours is a significant achievement in bone tumour care; however, not without complications. Neurovascular injury, muscle weakness and PJIs have a predilection for the lower limb, while prosthetic instability is likely following proximal humerus resection and replacement joint. Early presentation and meticulous soft-tissue preservation could reduce our complication rate.

Contribution

The findings highlight our experience treating aggressive bone tumours with limb-salvage surgery in our clinical context.

## Introduction

The incidence of malignant bone tumours remains relatively low, although still associated with serious medico-legal implications from complications [1,2]. Fortunately, the treatment for musculoskeletal tumours has evolved, with more patients now receiving limb salvage procedures over the classic options of limb amputation or palliation [3,4]. Surgery in combination with chemotherapy and radiation therapy in bone sarcomas is nowadays successful, with good oncological outcomes in more than 90% of all cases [4-7]. The use of neoadjuvant chemotherapy with its effective reduction in tumour size facilitates limb-salvage surgery with a reduced rate of local recurrence in primary malignant tumours in our setting [1,5]. Tumour volume in primary malignant bone tumours (PMBTs) like osteosarcoma is typically large at presentation in our context. Phillias et al. reported an average osteosarcoma tumour volume of 1114 cm3 at presentation [5]. Large tumour volumes at presentation are associated with mixed results concerning post-operative surgical outcomes [5,7]. Anecdotally, large tumours pose multiple challenges to the treating surgeon, both intra-operatively (i.e. prolonged operation times, bleeding, etc.) and post-operatively (wound healing and rehabilitation).

With advances in orthopaedic prosthetic technology, post-resection bone defects can now be successfully managed with modular tumour prosthetic devices [4,7]. However, with improved surgical limb salvage procedures, prosthesis-related complications remain a concern [3,4]. Equally, patients with musculoskeletal tumours in our context present at a late advanced stage of disease. Limb salvage in the latter surgical context is typically long and challenging and carries an increased risk of post-operative complications. Therefore, the primary objective of this study was to perform a retrospective clinical audit evaluating the nature, frequency, and distribution of surgical complications following limb-salvage surgery with endoprosthetic replacement for aggressive bone tumours at our specialised unit from 2017 to 2024. To our knowledge, there is no study similar to ours locally.

## Materials and methods

We conducted a retrospective review of patients who underwent limb-salvage for aggressive bone tumours at our tumour unit at Chris Hani Baragwanath Academic Hospital.

Patients above the age of 10 years who underwent limb-salvage procedures with prosthetic replacement were included in the study. Patients previously treated at a different unit and those who underwent revision surgery were excluded from the study. The medical records of all patients treated from 1st January 2017 to 31st December 2024 were perused.

Demographic data (age, gender, race), pathology factors (tumour diagnosis and limb affected), and surgery factors (documented complications) were retrieved and recorded from admissions, theatre, and outpatient files for analysis. Data were managed with Microsoft Excel and stored on a password-locked computer accessed only by the researchers involved. Descriptive statistics and Microsoft Excel were used to depict our results.

Our study sample was too small to employ statistical tools. Permission was issued by the Wits Human (Medical) Research Ethics Committee, ethics clearance number: M240353. All identifiers were anonymised, and patient records were attached with study numbers to conceal sensitive information in line with Ethical codes and the Protection of Personal Information Act (POPIA). 

## Results

We had a total of 20 patients who met the inclusion criteria. Female patients were slightly more than male patients, 11 (F) vs. nine (M), respectively. Our cohort was predominantly African, accounting for 85% of the study population. All age groups showed an equal distribution of burden of disease; see Table 1 below. 

**Table 1:** Characteristics of the study cohort. Total cases = 20.

Characteristic	n (%)
Gender	
Male	9 (45)
Female	11 (55)
Race	
Black	17 (85)
White	3 (15)
^*^Age	31.6 ± 17.96
Age categories	
0 – 17 years	5 (25)
18 – 24 years	5 (25)
25 – 34 years	5 (25)
> 35 years	5 (25)

Osteosarcoma accounted for 45% of diagnoses, followed by giant cell tumour (GCT) of bone at 30% (4/20). The other diagnoses were distributed equally and are shown in Table 2. The left and right sides of the body were equally affected, with 10 affecting either side. The lower limb was involved in 90% of the cases; see Table 3. 

**Table 2:** The diagnostic distribution of the burden of disease. Total cases = 20.

Diagnosis	n (%)
Osteosarcoma	9 (45%)
Giant cell tumour	6 (30%)
Chondrosarcoma	1 (5%)
Multiple myeloma	1 (5%)
Lymphoma	1 (5%)
Breast metastasis	1 (5%)
Inconclusive histology	1 (5%)

**Table 3:** Anatomical distribution of pathology by limb involvement. Total cases = 20.

Limb involvement	n (%)
Lower limb	18 (90%)
Proximal femur	7 (35%)
Distal femur	7 (35%)
Whole femur	1 (5%)
Proximal tibia	3 (15%)
Upper limb	2 (10%)
Proximal humerus	2 (10%)

A post-operative complication occurred in 70% (14/20) of our study cohort. Soft-tissue-related complications accounted for 20% (one hip abductor muscle weakness, one popliteal artery occlusion injury, and two peroneal nerve neuropraxias). Prosthesis-related events accounted for 35% of all complications. Table 4 depicts the total number of complications reported. 

**Table 4:** Tabulation and distributions of complications by percentage. Total cases = 20, Total complications = 14.

Complication	n (%)
Death	2 (10%)
Abductor weakness	1 (5%)
Peroneal neuropraxia	2 (10%)
Prosthetic joint infection	5 (25%)
Prosthetic instability	2 (10%)
Tumour recurrence	1 (5%)
Critical limb ischaemia	1 (5%)

The majority of the complications (78.4%) were in the age group 35 years and older; see Table 5. Neuropraxia (10%) and tumour recurrence (5%) were seen exclusively in those under the age of 18 years. Prosthesis-related complications such as prosthetic joint infection (PJI) and instability, as well as death, affected those above the age of 18 years, with the only vasculopathy-related complication affecting one of the oldest patients in the cohort.

**Table 5:** Complications burden by age group. Total cases = 20, Total complications = 14.

Age groups (years)	Abductor weakness	Peroneal neuropraxia	Prosthetic joint infection	Prosthetic instability	Tumour recurrence	Critical limb ischaemia	Death
0 – 17	0 (0%)	2 (10%)	0 (0%)	0 (0%)	1 (5%)	0 (0%)	0 (0%)
18 – 24	0 (0%)	0 (0%)	2 (10%)	0 (0%)	0 (0%)	0 (0%)	1 (5%)
25 – 34	0 (0%)	0 (0%)	2 (10%)	1 (5%)	0 (0%)	0 (0%)	0 (0%)
> 35	1 (5%)	0 (0%)	1 (5%)	1 (5%)	0 (0%)	1 (5%)	1 (5%)

## Discussion

Aggressive bone tumours are uncommon in our setting; however, late presentation with large tumours is the norm and poses a significant surgical challenge with an increased risk for complications related to limb-salvage procedures. Post-operative complications can be classified as either soft-tissue-related and/or prosthesis-related. Soft-tissue complications in the form of surgical site infection, haematoma formation, and muscle weakness are known to delay patient recovery and contribute to poor surgical outcomes [3,6]. Infective complication rates are variable following limb-salvage; however, they are consistently reported as the commonest reason for a need for secondary amputation [4,6,8-10]. Farid et al. reported a 4% infection rate, with two out of 52 cases developing infection following proximal femoral resection and tumour prosthesis replacement [8]. Infections were recorded in 25% of our cases in the form of PJIs. Our relatively high infection rate can be secondary to prolonged surgical times for large tumours, in a background of an already immunocompromised patient physiology. Deep infections ranged between 9% and 18% in segmental vs total femur replacements post-tumour replacements, supporting findings that longer procedures may increase the risk of post-surgical infection [11-13].

Henderson et al. reported five types of tumour prosthesis failure, namely Type I: soft tissue failure, Type II: aseptic loosening, Type III: structural failure, Type IV: infection, and Type V: recurrence [11-13]. We observed three Type I, 5 Type IV, and 1 Type V failures in our cohort as per the Henderson classification, which accounted for 33% (3/9), 56% (5/9), and 11% (1/9), respectively, with no Type II and III failure types observed. Fortunately, catastrophic mechanical failures (prosthesis fracture and component dislocation) are rare in the literature [9,14]. Our Type 1 failures were in the form of proximal humerus prosthesis migration in two cases of proximal humerus resection and replacement, as well as one case of hip abductor muscle weakness. The proximal humerus prosthesis migrations were seen early in the unit's establishment. These were related to significant rotator cuff tendon resection and loss of depressor function. Lately, we pre-emptively perform a reverse total shoulder-type proximal humerus replacement to improve post-resection instability. In a recent systematic review by Ciaani et al., they noted that even in high-volume tumour units, humeral prosthesis instability can be as high as 32%, necessitating revision surgery in 10% of cases [15]. Globally, complications related to proximal humeral tumour resection and replacement can be as high as 45% [14].

Osteosarcoma was the most common pathological diagnosis for prosthetic replacement in our study, followed by GCT of bone. Osteosarcoma is consistently the commonest diagnosis in reviews of PMBTs in patients under 30 years of age, and just over 50% of our cohort was below 30 years of age [1,5]. For patients over the age of 30 years, chondrosarcoma typically ranks second; however, GCT of bone was the second most common tumour in our study [2,15]. The latter observation is because our study was looking at complications in patients treated for aggressive bone lesions with tumour resection and prosthetic replacement, and not purely those with PMBTs.

Limitations

Our study is limited by a small sample size. Although the study spanned a reasonable study period, we also note that for the years 2020 to 2021, due to COVID-19 restrictions, hospital functions were heavily restricted, which led to disruptions in patient referrals. The latter, as well as the rarity of aggressive bone tumours in general, limited our sample size. Secondly, our study was retrospective and depended on the quality of data already recorded. We recommend a follow-up study in 10 years to review results based on a larger study sample. 

## Conclusions

The use of limb salvage surgery with tumour prosthesis implants for malignant bone tumours is a significant achievement in bone tumour care, however, not without complications. Neurovascular injury, muscle weakness and PJIs have a predilection for the lower limb, while prosthetic instability is likely following proximal humerus resection and replacement joint. Complications seem to increase with age. Early treatment and meticulous soft-tissue preservation could reduce our complication rate.
